# Using Participatory Action Research to Redirect Tinnitus Treatment and Research—An Interview Study

**DOI:** 10.3390/jcm13113099

**Published:** 2024-05-25

**Authors:** Julia Kajüter, Gerko Schaap, Anneke Sools, Jorge Piano Simões

**Affiliations:** Department of Psychology, Health and Technology, University of Twente, 7522 NB Enschede, The Netherlands

**Keywords:** tinnitus, participatory action research, qualitative, interview study, grounded theory, triangulation of stakeholder perspectives

## Abstract

**Background:** Chronic bothersome tinnitus is a prevalent tinnitus subtype placing a high burden on affected individuals, economies, and healthcare systems. Patient and professional perspectives seem to be partly misaligned on how to improve tinnitus research and treatments in the future. This qualitative interview study was aimed at exploring, comparing, and stipulating the perspectives of different tinnitus stakeholder groups on ways of redirecting research and treatments to reduce patients’ suffering while accounting for challenges within these practices. **Methods:** This study used the participatory action research approach to facilitate the stakeholder involvement. Semi-structured online interviews including five participants (two tinnitus patients, two tinnitus researchers and medical specialists, one general practitioner) were conducted. Inductive grounded theory and the constant comparative method were used for data analysis. **Results:** Four categories for suggested research adaptations ((I) ethical patient involvement; (II) prioritising cure versus coping research; (III) funding; (IV) ethical publication) and six categories for suggested treatment adaptations ((I) ethical professional support; (II) patient involvement; (III) interdisciplinarity; (IV) professional tinnitus education; (V) clinical treatment guidelines; (VI) psychological treatment) were identified. Participants held partly similar priorities such as increasing pathophysiological and cure research. Differences between participants included, for instance, patients aiming for increasing patient involvement in tinnitus research and treatments compared to professionals arguing that the excessive focus on patients’ conditions might reduce the patients’ chances of habituating to their symptoms. **Conclusions:** Four action redirections for improving tinnitus research and treatment practices were defined: (I) facilitating communication between and within stakeholder groups, (II) increasing the reflective use of patient involvement, (III) increasing interdisciplinarity, and (IV) reducing barriers to receiving psychological treatment.

## 1. Introduction

Tinnitus is understood as the perception of sounds like ringing or clicking without a corresponding external stimulus. According to the latest North American clinical guideline, experiencing tinnitus symptoms for more than six months indicates a chronic condition, whereas bothersome tinnitus is indicated by a higher severity of symptoms and its burden on an individual to the extent that professional healthcare support is sought [1]. Approximately 14% of the worldwide population suffers from some kind of tinnitus [2], whereas 1% of the general population reports having bothersome and more severe symptoms [3].

Chronic bothersome tinnitus is one of the most clinically relevant and impairing tinnitus subtypes. Suffering from chronic bothersome tinnitus includes a prolonged severe mental and physical burden, often resulting in high mental distress [1], more, especially mentally based, comorbidities [4], and a significantly reduced health-related quality of life (HRQoL) [5]. Other than patients, healthcare systems that are required to provide additional clinical care and economies needing to compensate for patients unable to work are affected as well [1]. All these instances face additional financial burdens due to the complexity [6,7], longevity [7], intensity, and interconnectedness of chronic bothersome tinnitus with other clinically relevant conditions [8]. Therefore, building well-established research and support systems is vital to help reduce strains on patients and resultingly unburden healthcare services, economies, and society as a whole [6,9]. Given its high clinical and economic relevance, this study will focus on chronic bothersome tinnitus, which will be henceforth referred to as tinnitus.

Despite the need for adequate tinnitus research and treatment, current professional practices are far from optimal. For instance, current research often overlaps in that the efficacy of existing treatments, mostly self-management strategies, is repeatedly tested, while aspects like pathophysiology, cure possibilities, and patient perspectives on relevant research topics are still only limitedly addressed [9,10,11]. Lack of funding and low professional involvement in tinnitus research are additional constraints to further progress in the field [9]. Moreover, due to the condition’s high heterogeneity and the incomplete understanding of its underlying pathophysiological mechanisms, no effective symptom-reducing or alleviating treatment for the phantom perception of tinnitus has been found yet. This significantly limits the abilities of medical professionals to offer adequate medical support to patients [9]. Given this prevailing lack of effective medical treatments [9] and the negative impact of tinnitus on patients’ HRQoL [5], current psychological treatment approaches largely focus on helping patients cope with and habituate to their tinnitus perception [12]. Yet, considering the condition’s high heterogeneity, such treatments are also only limitedly effective, whereas, for some patients, no option works at all [3,9]. Combined with the time commitment and financial costs of seeking professional aid, this leaves many patients with the sole option of self-managing their tinnitus, which can make them feel marooned and even more overwhelmed with their condition [13]. 

The complexity of problems connected to tinnitus research and treatment makes detecting and prioritising issues that prevent patients from improving their condition essential. Including patient perspectives in such investigations can help enhance the research’s relevance [14,15], quality [16], and effectiveness in reducing patient suffering [17]. Moreover, incorporating professionals such as researchers and clinicians in such research aims to set up realistic expectations and limitations for reaching these goals. Comparing the perspectives of these different stakeholders can increase the awareness of the current discrepancies between their priorities, perceived roles, and perceived limitations [18,19] while aiming to find ways of stipulating them, with the primary aim of improving patients’ conditions with the means currently available. 

One framework of such multi-perspective research is the participatory action research (PAR) approach [20]. PAR is an action-oriented approach that aims to constantly involve affected stakeholders in the process of tackling current public health issues [21]. In PAR, four iteratively repeatable stages can be distinguished. In Stage 1 (Problem Definition), the stakeholders and researchers involved in the study establish and define the issue on which the research will be based. In Stage 2 (Action), actions are determined and executed to deal with and reduce the problems connected to the issue at hand. Stages 3 (Observation) and 4 (Reflection) then focus on observing and reflecting on the effects the executed actions had on solving the previously defined issue. Based on these reflections in Stage 4, the problem can be redefined, and Stages 1 to 4 can be repeated as often as needed or feasible within the research [21]. 

PAR has already been increasingly implemented in projects aiming at improving mental health services with and for patients. Such studies have highlighted the importance of PAR in increasing patients’ wellbeing, feelings of empowerment and control over their condition, increasing patient-tailored professional support, enhancing the stakeholders’ knowledge and awareness of the investigated health issue, facilitating the exchange of experiences and perceived roles between and within stakeholder groups, as well as ameliorating the research’s relevance in helping improve patients’ conditions [20,22,23,24,25]. Despite this evidenced effectiveness of PAR in tackling public health issues, its application to the context of tinnitus and other neurological or audiological conditions is still largely missing. Given the previously outlined professional struggles and patients’ dissatisfaction with tinnitus practices [26], such an approach would, however, be crucial to prioritise research on topics that are relevant for tinnitus patients to reduce their suffering.

**Figure 1:**
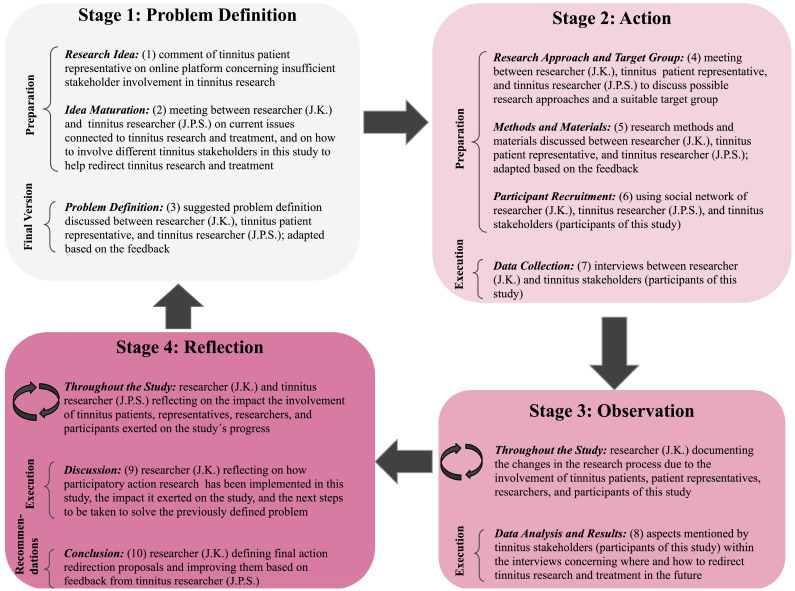
The 10 Steps of using the Participatory Action Research approach throughout this study. *Note.* This figure represents how the ten steps (*Research Idea*, *Idea Maturation*, *Problem Definition*, *Research Approach and Target Group*, *Methods and Materials*, *Participant Recruitment*, *Data Analysis and Results*, *Discussion*, *Conclusion*) were implemented within the four stages (*Problem Definition*, *Action*, *Observation*, *Reflection*) of the participatory action research approach. J.K. = abbreviation of the researcher and first author of this paper; J.P.S. = abbreviation of the tinnitus researcher and senior author involved in this study.

In light of the current challenges connected to tinnitus practices, this pilot study aimed to detect, compare, and stipulate the research and treatment priorities of individuals personally or professionally affected by tinnitus, as well as to formulate action proposals for redirecting current research and treatment practices to help improve patients’ conditions in the future. These action redirection proposals could involve the establishment of new actions or the alteration of existing ones within current tinnitus practices. To enhance the quality of research and its relevance for tinnitus stakeholders, the PAR approach was used as a framework throughout this study (Figure 1). 

The following main research question was addressed within this paper:

- According to stakeholders personally or professionally dealing with chronic bothersome tinnitus, where could its research and treatment be adapted to help improve patients’ conditions?

To answer this main question, two sub-questions were discussed:

- What are the differences and similarities between the stakeholders’ perceived issues, priorities, and suggested adaptations for redirecting chronic bothersome tinnitus research and treatment?
- How could the stakeholders’ suggested adaptations be stipulated in action redirections to help improve patients’ conditions?

## 2. Methods and Materials

The execution and documentation of this study took place between February and July 2023. A qualitative, semi-structured, online interview approach was used for the data collection as it helped to gather rich, deep, and exploratory insights into individual perspectives on the investigated issue [27,28] while eliminating geographical restrictions to participating [29]. This ensured that a wider population of tinnitus stakeholders could participate. This study was based on a constructivist point of view, meaning that we explored multiple individual perspectives and realities of tinnitus stakeholders and tried to combine them to establish commonly accepted solutions for the issue at hand [30]. 

The Standards for Reporting Qualitative Research were used as a guideline for writing this report to enhance its quality and transparency [31].

### 2.1. Participant Recruitment

Participants were recruited between March and May 2023. Snowball sampling was used as the sampling method [32]. As part of the recruitment procedure, two authors of this study (J.K. and J.P.S.) as well as the participants themselves contacted individuals in their social networks who fulfilled the inclusion criteria. Since this pilot study did not receive financial funding and due to the limited recruitment and data collection timeframes, this sampling procedure was evaluated as the most fitting.

To be eligible for this study, individuals needed to (a) deal with tinnitus in their personal and/or professional lives (e.g., patients, patient representatives, clinicians, researchers), (b) be at least 18 years old, and (c) be proficient in either the English or German language. For the final sample, it was desired that at least one representative of each stakeholder group (patient, professional) participated in this study.

The final sample consisted of five individuals who agreed to participate and for their data to be used for this research. Ages ranged between 37 and 68 years (Mdn = 43, IQR = 20). Two participants identified as female and three as male. Two participants were tinnitus patients and representatives of a patient organisation, one participant worked as a general practitioner (GP), and two were tinnitus researchers and medical specialists, one practising as an audiologist and one as a neurologist. Both patients had been suffering from tinnitus for five years and had no additional neurological, psychological, and/or audiological comorbidities. The professionals had been working in their fields for between 15 and 32 years (Mdn = 15, IQR = 17). One participant had a university degree, whereas the other four participants held doctoral degrees.

### 2.2. Materials 

#### 2.2.1. Demographics Form 

A document containing questions about the participants’ demographic data, including their age, gender identity, tinnitus background, amount of time being personally or professionally affected by tinnitus, highest academic degree, and current or last occupation was created. The form was available in English and German versions.

#### 2.2.2. Interview Questions, Probes, and Interview Slides

To enable consistency between the interviews with the different stakeholder groups, two sets of six interview questions were established (Table 1). One set of questions was designed for the interviews conducted with tinnitus patients, whereas the other set focused on the interviews held with the professionals. The questions were developed so that they dealt with the same overarching issue within both question sets (e.g., Question 4: tinnitus management within the participants’ tinnitus background; Question 6: wishes to improve tinnitus practices in the future). This aimed to facilitate both the comparability of data between the different stakeholder groups and the gathering of unique and individual information tailored to the participants’ varying tinnitus backgrounds. A combination of open- and closed-ended questions was used so as not to overwhelm participants with too many answer options while still encouraging them to share their own experiences as much as possible [33,34]. 

Other than the interview questions, two sets of probing questions were created for patients (Appendix A) and professionals (Appendix B) which could be asked during the interview to deepen the understanding of a certain issue under discussion [35].

To establish a structured but flowing conversation, interview slides were designed. The slides included an introduction of the researcher and this pilot study, the acquisition of the participant’s consent, six slides, each entailing one of the six interview questions and its probes, and a closing statement of the researcher. The slides were designed to help participants refer back to the questions and probes whenever needed and to let them decide whether to answer a question based on their first associations or, especially when being unsure about which topics to address, look at the probes provided. Particularly for patients for whom this might have been their first participation in a scientific study, the slides were intended to make them feel comfortable during the interviews. The presentation slides were available in English and German. 

### 2.3. Procedure 

All interviews were conducted between April and May 2023 using Microsoft Teams (version 22027.1100.1170.132). This software was selected because of features such as automatic transcripts and secure data storage (See Section 2.4.1). Each interview was held between the first author/researcher (J.K.) and one participant. In some cases, the participants and the researcher had already been familiar with each other beforehand. One day before each interview, the participants were digitally sent the demographics form and a link to a Microsoft Teams meeting. Filling in the demographics form beforehand helped to reduce possible distractions during the actual meeting. 

At the start of each meeting, the participants were offered to turn their cameras on or off based on personal preference to make them feel comfortable sharing more personal insights in the interview [36]. The researcher’s camera was always turned on. With this, establishing rapport, for instance, via minimal encouragement such as nodding or smiling was aimed at encouraging participants to share their ideas on the posed questions [37,38]. Throughout the meeting, the researcher shared her screen with the predesigned interview slides. At the end of the interview, participants were asked if they would like to receive their interview transcript, video, or the research paper once finalised to keep them involved and informed on the research beyond its data collection if desired. 

The main interview started with the researcher asking the six main interview questions and the probes fitting the interview. There was no predetermined time for the participants to answer each question. This was aimed at ensuring that all aspects deemed relevant by the participants could be discussed without time constraints. If the professionals were also suffering from tinnitus or the patients were professionals too, the interview questions for professionals were used, whereby probes about their personal circumstances were asked. With this, both their perspectives could be respected. To stimulate a wider variety of aspects discussed, the researcher emphasised throughout the procedure that the probes were solely example ideas and that the participants were free to choose other topics they wanted to talk about. The researcher asked follow-up questions to gain more in-depth insights from the participants on topics evaluated as relevant [28,38]. The researcher summarised her understanding of the participants’ main statements when considered necessary to ensure a correct understanding and/or encourage more elaboration on a topic [39]. After all questions had been discussed, the participants were asked for additional remarks and questions regarding study- or interview-related issues.

### 2.4. Data Analysis

#### 2.4.1. Transcripts

Interview transcription and storage were handled by J.K. Based on the guidelines of the University of Twente [40], after each interview, the recordings and the final pseudonymised transcripts were deleted from the researcher’s Microsoft Teams application and stored on a secured Google workspace. The recordings and transcripts were deleted in August 2023 from the researcher’s device. The transcripts were then handled by J.P.S. and stored and archived on the University of Twente’s secured Areda application for ten years [40]. 

To establish the transcripts, the researcher read through the automatically generated Microsoft Teams transcripts while simultaneously listening to the interview’s audio recording. Parts that did not correctly reflect what was said were adapted. Aspects such as coughs and high amounts of stutters that did not contribute to gathering the meaning of statements were either completely deleted or kept in small amounts when they were considered to reflect, for instance, the participant’s uneasiness or uncertainty about what to say. Factors such as irony and laughs which were solely perceivable with the audio and contextual background of the interview were added in brackets to ensure a correct understanding of the situation when reading through the transcripts [41]. Personally identifiable information such as names and dates were replaced with an *X* to ensure the participants’ anonymity [42]. Moreover, the transcripts were pseudonymised, meaning that each participant was linked to a reference number which enabled them to be referred to anonymously within this paper [43]. After having finished each interview’s transcription, the documents were checked again for accuracy and anonymity. 

#### 2.4.2. Developing the Coding Scheme 

Data analysis and the establishment of codes, themes, and categories were handled by J.K. To simplify the management and comparison of data across transcripts, the computer software ATLAS.ti version 9 was used [44]. 

Using the inductive grounded theory approach, codes, themes, and categories were newly established for each interview based on the unique information each transcript provided [45]. For each transcript, relevant excerpts were openly coded based on semantic and contextual meaning. The codes were then combined under one theme name if they entailed the same overarching topic [46]. For instance, codes in which patients addressed that professionals might have difficulties accepting feedback on their research practices which could complicate patient involvement in tinnitus research were combined under the theme name *limitations of patient involvement in research*. 

Using the constant comparative method [45], the established codes and themes of all five transcripts were investigated concerning their relevance for answering the research questions. The relevant themes were merged or adapted, resulting in the establishment of new themes or an increase in the number of codes defined under one theme. As an example of the former, the themes *importance of increasing professional tinnitus education* and *limitations of increasing professional tinnitus education* derived from two different transcripts were combined under the new theme name *perspectives on increasing professional tinnitus education*. Concerning the adaption of theme names, for instance, aside from the code referring to some professionals possibly having difficulties dealing with patients’ feedback on their research practices, other transcripts included codes about professionals worrying that patients might focus too excessively on their condition if they were involved in many tinnitus research projects, which could result in a worsened condition. These codes were then combined under the already established theme name *limitations of patient involvement in research*. 

Lastly, newly established or adjusted themes that addressed a similar issue were merged into one category [46]. For instance, the theme *limitations of patient involvement in research* was combined with two other themes (*importance of patient involvement in research* and *ways of implementing patient involvement in research*) under the category of *ethical patient involvement*. To finish this step, it was desired that all categories and their themes addressed different thematic aspects of answering the research questions. Some codes fit multiple themes and categories and solely using codes that fit one theme or category seemed to result in losing important data. This also showed how highly interconnected the issues connected to tinnitus practices were. Consequently, codes were excluded from this requirement. One example of a code used multiple times was not letting patients focus too much on their tinnitus condition to help them become habituated to their tinnitus perception. This code was used in the themes *limitations of patient involvement in research* (category: *ethical patient involvement*) and *limitations of professional support* (category: *ethical professional support from the beginning*). 

To deal with data saturation, the researcher asked the participants for additional remarks and feedback on the interview questions after each interview. This ensured that no topic that participants deemed important was dismissed. Moreover, the researcher read through the determined codes, themes, and categories after each coding step and adapted them if considered necessary. The next coding step only followed when no adaptations were made during the last time the codes, themes, and categories were reread. To deal with intersubjectivity, the codes, themes, and categories were, once finalised, reviewed by the other three authors/researchers of this paper (J.P.S., G.S., A.S.) and feedback was discussed and incorporated.

### 2.5. Researcher Characteristics and Reflexivity

J.K., who mainly executed and documented this research, conducted this study within her training to become a health psychologist. Therefore, J.K. still had limited experience with conducting qualitative studies and had not engaged in tinnitus research before this study. As a result, this study might have been handled differently by researchers who were more experienced in qualitative research and tinnitus studies. However, not having conducted tinnitus research before and not being a tinnitus patient herself also offered J.K. the possibility of conducting the interviews as a mediator between the different stakeholder groups instead of being directly involved in the investigated issue. Nevertheless, being a researcher for this pilot study might have implicitly made J.K. relate more to the situation the interviewed researchers were in. This could have influenced the reporting of this study in that the documentation might have been focused more on adapting to what the researcher stakeholder group addressed compared to what the patients or clinicians mentioned. To counteract this possible bias, J.K. consistently exchanged feedback with tinnitus stakeholders and the other three authors/researchers involved in this study (J.P.S., G.S., A.S.) who were more experienced in conducting qualitative, tinnitus, and PAR research at different stages throughout this pilot study. All four researchers involved in this study hold a psychology degree with specialisations in different areas. One result of this high number of psychology researchers involved might have been that a higher focus was put on discussing ways of enhancing patients’ HRQoL and facilitating tinnitus management within the set-up, the interviews, the analysis, and the documentation of this study. This could have directed participants towards revealing more information about these psychological topics, which stands in contrast with medically oriented researchers, whose focus might have been more on reducing the acoustic perceptions and bodily factors associated with tinnitus. Therefore, implementing the PAR approach, including a diverse group of tinnitus stakeholders, and establishing direct and constant supervision of more experienced researchers with different backgrounds (J.P.S., G.S., A.S.) was aimed at gaining a more multidisciplinary view and gathering diverse feedback on points of improvement throughout this study to reduce researcher bias. Based on this constant exchange, no conflicts of interest between the involved researchers and stakeholders were detected throughout this pilot study.

## 3. Results

Interview lengths ranged between 60 and 100 (Mdn = 75) minutes. Four categories for the suggested research adaptations were identified: (I) ethical patient involvement, (II) prioritising cure versus coping research, (III) establishing realistic opportunities to receive and keep funding, and (IV) ethical publication. For the suggested treatment adaptations, six categories were established: (I) ethical professional support from the beginning, (II) time-efficient versus adequate patient involvement, (III) increasing interdisciplinarity while respecting organisational challenges, (IV) offering professional tinnitus education, (V) adapting and increasing awareness of clinical treatment guidelines, and (VI) facilitating the reception and seeking of psychological treatment. If professionals’ opinions differed, they were differentiated based on their specialisation (GP versus researcher/medical specialist (audiologist versus neurologist)). If opinions were similar, they were collectively referred to as professionals. Due to patients’ similar tinnitus backgrounds, they were continuously collectively referred to as patients. The participants’ reference numbers were used to refer to them anonymously. PA referred to patient, PR to professional.

### 3.1. Research Adaptations

#### 3.1.1. Ethical Patient Involvement 

This category discusses participants’ opinions on risks and chances of patient involvement in tinnitus research, as well as suggestions on how to increase the involvement of tinnitus patients in research studies. The identified themes are *importance of patient involvement in research, ways of implementing patient involvement in research,* and *limitations of patient involvement in research*. 

##### Patient Perspective

Both patients deemed the involvement of patients in tinnitus research to be crucial to establishing actual improvements in scientific practices and patients’ conditions. Hence, without it, they feared that research would target irrelevant topics that were not related to finding a cure and did not help them improve their condition. Despite this perceived importance of patient involvement and their perception of patients as motivated to participate, both patients perceived current tinnitus research to be too focused on professional perspectives, giving patients too few opportunities to share their expertise, experiences, and ideas. To increase the involvement of patients in tinnitus research, they suggested that researchers could contact patient-led tinnitus initiatives to request feedback on their research ideas. However, patients also found that this was often complicated since, from their experiences, some professionals had difficulties dealing with patients’ criticism on their practices:

“*I think the hard part is dealing [with feedback]. It means you also have to open yourself up to criticism and I think a lot of researchers say that they are open to that but when it actually happens, they’re very, very upset.*”

(PA1)

Such difficulties were perceived as impeding the establishment of relevant tinnitus research and increasing patients’ dissatisfaction with the current situation. 

##### Professional Perspective

Similar to the tinnitus patients, the professionals perceived tinnitus patients as being willing and motivated to participate in research. They also indicated that involving patients in research was crucial for detecting their priorities, achieving actual improvements in their conditions, and ensuring the research’s relevance. On the other hand, limitations of this strategy were mentioned. One professional explained the challenges as follows:

“*I think that fundamentally the tinnitus patients are looking for a solution and they are very famous for being willing to go through almost any therapy you can think up even on the verge of quackery. If you can instil in them some kind of hope that it might help, they’re really suffering and they will do anything to improve their tinnitus which also makes them more vulnerable to people wanting to take advantage of them for taking this medication, this laser therapy, or these magic magnetic waves. Lots of people will take advantage of them or lots of them would be willing to be taken advantage of if there’s a small sliver of hope that they could be helped.*”

(PR3)

Hence, the professionals seemed to be more cautious in involving tinnitus patients in their research since they did not want to place any further burden on the patients and seemed to consider it to be in their responsibility to prevent tinnitus patients from suffering any further harm. Based on the professionals’ perspective, the risks and chances connected to patient involvement in tinnitus research needed to be carefully and ethically balanced, whereas patients focused more on trying out new possibilities to help research move forward despite the risks this might entail.

#### 3.1.2. Prioritising Cure versus Coping Research

This category discusses participants’ opinions on the topics future tinnitus research should increasingly focus on to help improve patients’ conditions. The themes are *importance of cure research*, *ways of facilitating cure research,* and *limitations of cure research*.

##### Patient Perspective

The patients stated that the main focus of research should be on finding a cure or at least treatments reducing the acoustic perception of tinnitus. However, they felt that current research constantly focuses on finding ways of better coping with tinnitus, which was not in their interest:

“*Achieving a cure should be the ultimate aim of research. I say ‘should be’ because there’s a lot of research that’s actually not aimed at a cure, but it’s aimed at yet another coping or management strategy where I feel like ‘We already have this’.*”

(PA1)

To find a cure, patients argued that research should be more focused on discovering tinnitus’ pathophysiology and the underlying mechanisms influencing the condition. 

##### Professional Perspective

The professionals agreed that finding a cure would be the ultimate aim of research since all other treatments would not be needed anymore and burdens on patients and professionals would be reduced. However, other topics such as enhancing current management strategies should not be neglected either, as they could help patients to better deal with their tinnitus while waiting for the cure:

“*I think a cure may well still be somewhere off and if you only focus on that, you miss opportunities to help people in the ways that are possible at the moment. But if you’re only focused on psychological help and don’t pursue a cure, you’ll never get people to the endpoint. Really, most [tinnitus patients] want to be done with their tinnitus so it needs both. […] I think it’s very, very difficult to say one is more important than the other or negate the need for the other.*”

(PR1)

The professionals agreed with the patients that, to develop a cure and find symptom-reducing treatments, more research should be conducted on tinnitus’ pathophysiology and its underlying mechanisms. Cure research should be enhanced by increasing motivation and interest in researchers from different disciplines and giving them the freedom to explore tinnitus from the field they wanted. One problem with this complex and time-intensive research was that researchers still wanted and needed to publish scientific work. Therefore, based on the pressure to regularly publish, many researchers would currently test tinnitus treatments solely based on a guess that they might be effective and without supporting evidence behind it. This often leads to insignificant and rather unhelpful results, the time spent on which could have been invested in building solid background knowledge on tinnitus:

“*We need more foundational understanding of what tinnitus is and what tinnitus does. Only when you understand what’s broken can you come up with theories of what you could do to affect these broken things in a more positive direction. […] until we don’t know what exactly breaks, you can’t develop a test for applying treatments. But that’s what we do in research, because otherwise, I couldn’t publish. So what do you do? You guess. […] And then you see not really effective tinnitus treatments.*”

(PR3)

Hence, on the one hand, the patients prioritised research on cure options and underlying mechanisms of tinnitus, whereas the professionals struggled to prioritise one over the other. 

#### 3.1.3. Establishing Realistic Opportunities to Receive and Keep Funding 

This category discusses participants’ perspectives on the role of funding and on how to increase the possibilities of receiving funding for tinnitus research. The identified themes are *importance of increasing funding*, *ways of increasing funding*, and *obstacles to increasing funding*.

##### Patient Perspective 

Increasing financial support for tinnitus research was seen as very important to improving tinnitus research by the patients. According to the patients, this could be achieved by establishing objective measures for tinnitus, as this would make the condition and treatment improvements more measurable, desirably attracting more (e.g., pharmaceutical) companies to financially support research.

##### Professional Perspective 

Professionals also perceived the lack of funding as a central hindrance to executing important tinnitus research projects. This was stated to be partly due to the lack of benefits institutions would see to establishing research on tinnitus compared to the research on other illnesses:

“*Since there is little money to be earned here [tinnitus research], the research funds of the industry are of course much lower than, for example, with a blood lipid reducer or other heart medicines, that first of all a billion research funds are invested by the company, or also with cancer patients a billion euros were invested in research without any problems, in the hope, however, of developing medicine that is very promising and can be marketed well and brings high profits, so here with tinnitus, with the currently insufficient therapy approaches, the profit margin for the industry is low. That is why there are hardly any industry-sponsored studies.*”

(PR2)

This lack of financial support was also the reason why only a limited number of researchers investigated tinnitus and why advancements in research were partly lacking:

“*[…] you can’t expect just based on the area of work that you’re going to get a sufficient amount of funding to do the complex tasks that you have in front of you. And so the quality of much tinnitus research is very, very low because if you didn’t produce a lot, and if you didn’t produce positive, significant results, you’re going to lose your funding. And so what do you do? You either say, well, I’m getting out of the tinnitus business because I can make more progress somewhere else or you’re risking doing things that actually aren’t good for making progress in the area of tinnitus.*”

(PR3)

Another problematic factor was that, to increase the chances of funding, researchers needed to make large claims that were often not scientifically proven. These studies often had negative results which, if published, would most likely make the researchers lose their funding. Hence, the kinds and amounts of conducted research were influenced by the amount of funding, whereas the amount of funding was again influenced by the significance of research results, which put researchers in a vicious cycle:

“*What you risk is that if you want money in tinnitus, you’re going to be inclined to make claims that are maybe bigger than the evidence basis that you have and if you don’t have positive results to not present those negative results as loudly as you probably should because then you risk that you’re gonna reduce your future funding. So, if we’re completely honest with the way we do research, then we’re shooting ourselves in the foot.*”

(PR3)

Both stakeholder groups therefore evaluated an increase in funding as an important factor in improving tinnitus research and desirably relieving the burden on tinnitus patients thanks to newly established findings. However, whereas patients primarily addressed attracting commercial or pharmaceutical companies in investing in tinnitus research, professionals also outlined the pressure and difficulties researchers faced in attracting and keeping funding due to the complex topics that needed to be addressed within tinnitus research and the resulting time-consuming nature of such projects, which often did not receive the financial support they needed. This shows the similar wishes of both stakeholders but also illustrates the clash between patients’ expectations and researchers’ experiences of how to successfully attract and keep funding.

#### 3.1.4. Ethical Publication 

This category focuses on how the number and the content of published tinnitus studies could influence patients’ tinnitus conditions. The defined themes are *importance of publication* and *obstacles to publication*.

##### Patient Perspective 

Patients reported that, despite their acceptance of self-managing their tinnitus due to the current lack of successful treatments, they wanted to see that actions by others were nevertheless taking place. One possibility of such actions was determined to be the establishment of a high number of publications on tinnitus research:

“*I’ve always said when there’s research, there’s at least hope. So that [seeing that new tinnitus research articles were published] for me was an important thing.*”

(PA2)

For the patients, seeing new research on tinnitus showed them that professionals were trying to help and support patients with their condition, making them feel less alone with their tinnitus. From the patients’ perspective, more tinnitus research could still be published by professionals.

##### Professional Perspective

The professionals also expected that, for patients, just knowing that research was taking place made them more hopeful about their condition. However, one participant also reported struggling between showing that research was taking place and making findings publicly available only when they would gradually improve patients’ lives so as not to trigger false hopes:

“*I personally don’t publicise my own results a lot because my viewpoint is I want to publicise them when we’ve found something that needs to get out there because it’s really going to change things […] So my approach is usually to sort of quietly get on with the research and then share it widely when it reaches a point where it needs to be.*”

(PR1)

Moreover, patients should not focus too much on finding every newly published paper on tinnitus, as this high focus on their condition could decrease their chances of habituating to their tinnitus perception. In addition, the dilemma between working on longer, more complex projects without being able to inspect other important topics for a longer time versus regularly publishing work which was only very limitedly advancing the field was addressed by the professionals. 

This shows the difference between patients’ wishes to know and access as much information on tinnitus as possible, whereas professionals rather seemed to want to prevent patients from becoming too focused on their tinnitus and on the possible false hopes tinnitus research could elicit. This poses the question of whether to share as much tinnitus research as possible while risking triggering false hopes in patients or to balance the number of publications so that hope was induced but inducing false hope in patients was avoided.

### 3.2. Treatment Adaptations

#### 3.2.1. Ethical Professional Support from the Beginning 

This category focuses on the participants’ perspectives on patients receiving professional support to improve their tinnitus condition. Themes are *importance of professional support, experiences with professional support, ways of increasing professional support, limitations of professional support,* and *ways of dealing with limitations of professional support*. 

##### Patient Perspective

The patients wanted to be more informed about the possible development of their tinnitus and receive more professional support in dealing with their condition. Especially in the beginning, they regarded this as very important since no one seemed to prepare them for how their wellbeing and symptoms would possibly develop or what they could do to deal with their condition. Both patients felt like they did not receive this kind of support at the start of their tinnitus condition. They argued that having some kind of “ally” would have helped them feel less distressed in the beginning:

“*I was just kind of trying to work this through myself. So again if that could have been avoided, boy, that would have been a big help […] [Somebody who] lead[s] you through the maze of getting to the point where you’re habituated and keeps encouraging you that it’s going to happen. That’s where I think the need is right now.*”

(PA2)

Moreover, the patients reported wanting to be able to attend more organised informational sessions or workshops about the progress of and current knowledge on tinnitus research and treatment options. This could help them to discuss, explore, and understand their condition and possible treatment approaches. However, they also knew that such informational sessions had already been organised before but since research was not evolving much, they were cancelled after some time. 

##### Professional Perspective

The professionals also felt that informing patients about their condition’s background and possible progress could make a significant difference in patients’ tinnitus-related distress and HRQoL. One important factor they saw was expressing their experiences of how conditions usually improved. Nevertheless, one still needed to stay realistic:

“*I think an overly positive view can be almost as harmful as an overly negative view. […] at least for the majority of people with tinnitus, an optimistic view or a fairly optimistic view can help. It’s not misleading to say most people’s tinnitus symptoms do improve but it takes a long time for them to improve. It’s not quick even where it does happen it can take many, many months or even years.*”

(PR1)

They further recommended differentiating between mild and more severe cases: providing basic information on tinnitus is often enough for mild cases, but severe cases have fewer improvement options and might require extra psychological treatments. Moreover, professionals should educate patients on the danger of seeking too many treatments and focusing too extensively on the physical and psychological burden connected to their condition as this prevents the brain from getting used to the symptoms and becoming habituated:

“*The act of seeking treatments for the tinnitus sound itself gives a very strong signal to the brain to keep monitoring the tinnitus and to keep treating it as important and as a threat. I worry that the act of seeking treatment damages and hinders habituation, which we know does work for most people. So, I think a lot of my advice to people is, yes, the research is happening but please let it happen, and don’t go seeking out the very latest treatments in every applicable option, because I think all it’s going to do is harm.*”

(PR1)

To conclude, the importance of receiving adequate support and background information on tinnitus, especially at the onset of patients’ tinnitus condition, was emphasised by both stakeholder groups. Professionals again expressed their concerns about offering any possible treatment to patients but also agreed that seeking support from different specialists to discover possible underlying factors of patients’ tinnitus was important. Supporting and informing patients throughout these treatments was pivotal for all stakeholders, whereas patients reported that they did not always experience this in their own past tinnitus treatments.

#### 3.2.2. Time-Efficient versus Adequate Patient Involvement

This category discusses the participants’ opinions on actively involving patients and their needs in their clinical tinnitus treatment. The identified themes are *importance of patient involvement in treatments* and *limitations of patient involvement in treatments*.

##### Patient Perspective

According to the patients, every treatment should be patient-centred. They also wanted their GPs to acknowledge their condition more, even if just briefly, to feel understood and validated. The patients often found that this was lacking based on their personal experiences:

“*[…] these old fossil type of doctors who believe that they know what’s best for you and they’re going to tell you what to do kind of thing […] when I go to my primary care physician now, it’s about getting labs done and getting tests and things. Tinnitus has never been brought up. It’s never discussed. So it’s kind of ignored.*”

(PA2)

##### Professional Perspective 

Professionals confirmed they knew that patients wanted to be asked about their treatment needs. However, due to their currently limited treatment possibilities, one medical specialist addressed the fact that focusing too much on satisfying patients’ needs took away the possibility for patients with other illnesses to receive effective and symptom-reducing treatments. Therefore, he instead aimed to send such patients to psychologists or psychiatrists to help them cope with tinnitus:

“*Given that you can’t cure the problem with medicine, then the goal would be to get them to the psychologists and psychiatrists who can try to cure them, maybe with medication, more commonly through therapy. So I don’t know what I would want my ENT [ear, nose, and throat doctor] to do. They’re wasting their time. They’re not talking to other patients whom they can maybe heal compared to if they’re talking to the tinnitus patient, whom they can’t heal.*”

(PR3)

Moreover, the professionals reported that current clinical practices had difficulties deciding between standardising treatments, hence, facilitating better research on their effectiveness, and increasing patient-centred treatment while neglecting possible research evolvements to be made.

As a result, whereas patients wanted to talk about and be involved in their tinnitus treatment irrespective of the medical professional they were seeing, due to their limited time and the high number of patients visiting their practice, some professionals saw the need to keep interactions informative but time-efficient. This might, however, contrast with what patients aimed for within their treatments.

#### 3.2.3. Increasing Interdisciplinarity While Respecting Organisational Challenges

This category discusses participants’ opinions on interdisciplinary tinnitus treatments. The themes are *importance of interdisciplinarity, ways of increasing interdisciplinarity,* and *obstacles to interdisciplinarity*. 

##### Patient Perspective 

As a general criticism on current tinnitus treatments, a patient mentioned that the current placement of tinnitus in the ear, nose, and throat (ENT) area should be loosened so that more cooperation between the different departments could take place. This would help with recognising tinnitus’ complex and heterogeneous nature.

##### Professional Perspective 

The professionals also supported the patients’ view. It was emphasised that more clinics and interdisciplinary treatment options should be established in which the patient would be treated in a more holistic way:

“*Obviously, a single approach with a focus on one aspect of tinnitus is not very promising, which can be seen in the fact that individual treatments only have a low chance of success. […] it certainly makes sense to have interdisciplinary therapy approaches and people from different specialist groups that try to work together and […] have more specialist outpatient clinics in which different doctors are who work in different specialist areas, including neurologists, psychologists […] in which we see the patient in his entirety and try to achieve an improvement.*”

(PR2)

One professional also addressed the option of having a fixed day on which patients could visit an interdisciplinary clinical setting and be checked in all medical and psychological areas. With this, the waiting time would be decreased and findings could be directly combined and discussed across disciplines. Yet, with the limited capacity of such clinics, only severe cases which generally have a lower chance of achieving a substantial reduction in their suffering could be addressed. Moreover, these interdisciplinary clinics would need to limit patients’ visits. Otherwise, they would consult them over and over again in the hope of finally detecting an effective treatment for themselves:

“*You can’t treat it [tinnitus] with a high degree of success and they [patients] will overrun our clinic if you let them because they’re suffering so much.*”

(PR3)

With respect to the previous statements, a general consensus on increasing interdisciplinarity in tinnitus treatments was established among all participants. On the other hand, the professionals also addressed the organisational challenges of establishing such interdisciplinarity, despite its dominant advantages, which needed to be respected when increasing the collaboration between disciplines. 

#### 3.2.4. Offering Professional Tinnitus Education

This category discusses participants’ perspectives on the current tinnitus education of professionals. The identified themes are *importance of professional tinnitus education* and *perspectives on increasing professional tinnitus education*.

##### Patient Perspective

Patients generally saw the need for professionals to be better informed about tinnitus because, from their experience, little was known by the ones they consulted:

“*Again, going back to my experience and I’ve heard this from so many people that your GP and ENT doctor and audiologists know almost nothing about tinnitus. […] at least improving that a little bit would already help.*”

(PA1)

##### Professional Perspective

Professionals expressed that during their medical studies, tinnitus was a very little discussed topic. Therefore, the GP agreed with the patients and expressed that he would like the focus on tinnitus to be increased:

“*I think that it [tinnitus] is certainly a sensible topic and should be in the advanced study area at least once in a study block […] to address this condition and therapy with the most current therapy approaches based on studies and data. I think that would make sense for many [medical] students.*”

(PR2)

On the contrary, a medical specialist perceived increasing the awareness on tinnitus in medical studies as not profitable since, at the moment, there were no insights that could substantially improve current care. Hence, research and more useful results needed to be achieved first, and then they could be addressed in medical studies:

“*While informing doctors is always nice, they’re informed of a tonne of stuff and at the end, they’re going to say ‘Well, what do I do differently? Because of what you told me, I will be kind, will give them my attention and send them to psychiatry.’ OK, but you don’t need to make that a push in medical education.*”

(PR3)

Comparing these opinions, stakeholder statements on whether to increase tinnitus education within the medical field were twofold, with the patients and the GP opting for more education, in contrast to the medical specialists coming from a tinnitus research background, who did not see the need to do so. This might illustrate the difference in the amount of tinnitus knowledge professionals have based on their occupational background. It might also show the varying perspectives of professionals on the extensiveness of their own tinnitus knowledge or the extent to which they share this knowledge with patients which could be connected to the often unsatisfying experiences patients had in their treatment encounters.

#### 3.2.5. Adapting and Increasing Awareness of Clinical Treatment Guidelines 

The following category addresses participants’ perceptions of current guidelines for clinical tinnitus treatments. The themes discussed are *importance of clinical treatment guidelines, experiences with clinical treatment guidelines,* and *ways of improving clinical treatment guidelines*.

##### Patient Perspective 

Patients offered the criticism that many of the professionals they consulted were not aware of the current treatment guidelines for tinnitus. To increase the research input, one patient suggested that the clinical guidelines should be changed to include registries in which clinical tests and assessments made on patients could be stored to have more data to assess and to conduct research upon:

“*[…] if there were some way of rewriting those clinical guidelines to include that there are registries where that [data of the] study could go and be evaluated because everything’s about big data now too. So if you have thousands of these things [data sets of studies] to look at, you know, maybe some pattern would emerge.*”

(PA2)

##### Professional Perspective

Consistently with the patients’ perspectives, the GP was not aware of current tinnitus treatment guidelines:

“*Here [country of residence], it is not known to me, but it would certainly make sense. That is what I know to be a weakness [of current tinnitus practices], that there is no clearly structured recommendation of diagnostics and therapy and between the therapy approaches for [different] types of tinnitus. That is not so common in X [country of residence]. But it would be helpful, in any case*.”

(PR2)

A medical specialist also mentioned that it was common that standardised, mostly old, and partly irrelevant guidelines were used and adapted in different ways by different professionals for assessments on tinnitus:

“*There are certain things in certain guidelines that don’t make any sense and everybody knows it, but they’re in the guideline. Maybe it’s an old guideline or maybe it’s just been passed down throughout the years and it’s just stayed in there because some powerful person believes in it. No one else does. That’s the foundation and then you adapt based on your own clinical experience as well as your capabilities.*”

(PR3)

Based on this, no stakeholder was completely satisfied with current tinnitus treatment guidelines. As a result, adaptations concerning, for instance, including study data in registries, establishing more relevant guidelines, or simply increasing public, and, particularly, professional awareness on such guidelines were aimed for.

#### 3.2.6. Facilitating the Reception and Seeking of Psychological Treatment 

This category discusses the participants’ current perspectives on offering psychological treatment to tinnitus patients. The themes are *importance of psychological treatment, ways of increasing psychological treatment,* and *obstacles to psychological treatment*.

##### Patient Perspective 

The patients reported that they perceived psychological treatment as being particularly important for newly developed tinnitus cases to help individuals cope with their condition. However, for the patients themselves, as they were already largely habituated to their tinnitus perception, psychological treatment was evaluated to be rather unhelpful:

“*I would have to be really, really convinced that this had something to offer to make me interested in it. However, I think the opportunity is for people who have newly developed it. I think there’s a great need there to provide them with that kind of hope, like we were talking about before. You know, if there are therapies and psychology that would integrate that kind of stuff into it or lead you through the maze of getting to the point where you’re habituated and keep encouraging you that it’s going to happen. Then, I think that’s where the need is right now.*”

(PA1)

On the other hand, patients criticised the fact that their experiences with psychological treatments were marked by long response times, directive instead of patient-centred approaches, and outdated practices:

“*It’s [psychological treatment] also very much like top-down. It’s not patient-centred, but it’s just telling the patient what they should be doing and how you should be thinking about your tinnitus instead of, you know, really working with the patient. So I don’t think that’s a good model for counselling. I think it should always be patient-centred. So yeah, it [personal experience with psychological treatment] wasn’t actually super helpful.*”

(PA1)

##### Professional Perspective

Especially since researchers are still learning about tinnitus and searching for a cure, the professionals deemed it important to improve psychological care for patients, even though this only helps with coping and not with reducing the perception. However, the professionals perceived many patients as holding prejudices and fears of consulting a psychologist. Therefore, many did not want to go to a “head doctor”:

“*[…] from my experience, in psychotherapy there are fears of contact [from tinnitus patients] since tinnitus is understood more as something organic or somatic. Many feel stigmatised by neurological indications and portrayed as mentally ill, whereas for them it is often a somatic illness.*”

(PR2)

One professional also indicated that, even though some patients expected him to, medical professionals were not there to provide the emotional support that psychologists offered. Therefore, he strongly recommended that patients sought support from psychological professionals. A way of circumventing direct psychological treatment would be the increased focus on providing care via online applications. These could serve the same function as psychological treatment but, for patients unwilling to got to in-person therapy, still offered an option of seeking such support in another way. Despite the proposal of circumventing psychological care, it was also stated that the first priority should still be to reduce the present stigma on psychological treatment. Moreover, increasing the physical and timely proximity with which patients received psychological treatment after having finished their medical assessments should be worked on:

“*And so if I can’t reduce the stigma, then I need to reduce the amount of friction required to get you into that person’s office, because if I can reduce friction, then, despite the stigma, […] I’m walking you to the door. I’m introducing you [to the psychologist] and I’m sitting you down, so I don’t really care what the stigma is.*”

(PR3)

Patients and professionals saw the importance of seeking psychological support when facing tinnitus symptoms. Nevertheless, using the advantages of psychological treatment still seemed to be difficult due to present stigmatisation or fear of such by tinnitus patients. Therefore, patients needed to be accompanied and supported by professionals to seek support.

## 4. Discussion

### 4.1. Summary of Findings and Similarities of Stakeholder Priorities

Using the PAR approach, this pilot study identified stakeholders’ priorities for improving tinnitus research and treatments. The primary aim among all participants of this study was to find a cure to reduce patient suffering. Other priorities included increasing the funding and publication of tinnitus-related studies and establishing more interdisciplinary research to better explore cure possibilities for tinnitus. Especially at the beginning of patients’ conditions, medical treatments should focus more on supporting and informing patients about tinnitus. To succeed in this, professionals need to have sufficient background knowledge on tinnitus and be aware of current clinical treatment guidelines to offer adequate support. Particularly for GPs, who are usually not confronted with tinnitus on a daily basis, knowing such guidelines and ways to further proceed in the treatment was evaluated as helpful to improve the professional care patients received. Moreover, more interdisciplinary treatment approaches, which also include psychologists, should be facilitated. Lastly, it was agreed that patients should be more involved in the establishment and execution of tinnitus research and treatments to increase the practices’ relevance and, as a result, help in improving their conditions. 

### 4.2. Differences in Stakeholder Priorities 

Stakeholder priorities differed mainly because professionals reported limitations in meeting patients’ needs, which were attributed to the current constraints on financial, time, and professional support for tinnitus research and treatments. Patients’ priorities were centred around increasing patient-oriented practices and establishing more research on cures instead of self-management. However, professionals addressed the fact that complex and cure-oriented studies were often not funded, leaving little possibility to investigate these topics. Moreover, they argued that high patient involvement might reduce patients’ chances of habituating to their tinnitus perception. Therefore, despite patients’ motivation to be more involved, professionals were hesitant to include patients in such research. 

Another priority for patients was to receive more professional support from clinicians throughout the progress of their condition, particularly at the beginning. The clinicians, on the other hand, indicated that their time constraints and lack of options to successfully reduce patients’ symptoms made them focus more on patients whose conditions they could actually improve. Moreover, they rather perceived that these needs for emotional support should be approached by psychologists instead of medical professionals. 

These examples illustrate the differing role perceptions and role expectations the patients and professionals participating in this study seemed to partly hold towards each other. The professionals seemed to see their role in protecting vulnerable and possibly unknowing patients from experiencing any further harm on top of their already distressing tinnitus condition. On the contrary, patients did want to be involved and saw their role in actively contributing and speaking up for themselves in research projects as they did not feel their needs and priorities were being properly represented by professionals. Both patients and professionals aspired to be in control of tinnitus practices due to different reasons (protecting patients and being experienced in tinnitus research and treatments, therefore knowing their limitations, versus perceiving professionals as not accurately representing their wishes and needs). The conducted interviews leave the impression that these stakeholder groups and their differing role perceptions have not been properly communicated with each other before, which seems to have led to misunderstandings and dissatisfaction, particularly among patients.

### 4.3. Establishment of Action Redirections

Based on the participants’ partly differing perspectives and priorities for action redirections, stipulations were made. The first main stipulated action redirection was defined to be establishing better communication among tinnitus stakeholders. This also includes the second redirection of reflectively increasing patient involvement in tinnitus practices. This means that possible issues (e.g., reduced chances of habituation) should be communicated to patients while then letting them decide the extent to which they seek treatment or engage in research projects. Otherwise, patients might be too focused on alleviating their symptoms, which then has the opposite effect of an excessive focus on and suffering from their tinnitus. Openly communicating needs and their limitations further aims to let stakeholders better understand each other’s actions and perceptions while collaboratively finding ways of satisfying both parties with the decisions made. This seems to be particularly relevant for interactions between patients and GPs or ENTs, with whom tinnitus patients reported the lowest communication satisfaction in a recent investigation published by Carmody et al. [26]. Meetings with audiologists, on the other hand, were evaluated as the most satisfying by patients [26]. Studies by Pryce and colleagues [47,48] already investigated ways in which better communication within tinnitus treatment could be reached. They opted for establishing basic therapeutic skills and shared decision-making for medical professionals in their practices to facilitate more patient-centred treatments. These skills were shown to enhance the treatments’ effectiveness and the patients’ satisfaction with them [47]. Moreover, designing Option Grids was argued to facilitate capturing patients’ preferences within clinical encounters and improve the communication of these [48]. Examples of implementing interactions between stakeholders within tinnitus research are the PAR approach applied to this study, the Core Outcome Measures in Tinnitus (COMiT’ID) initiative, in which stakeholders collectively mapped tinnitus research and treatment priorities [49], as well as the involvement of different stakeholder groups to design Option Grids for clinical encounters in a work by Hall and colleagues [48]. To execute such research projects and have the skills to mediate interactions between different stakeholders, some professionals might need further training from colleagues specialised in this research area. As reported by Yu et al. [50], such programmes or workshops targeted at training researchers in involving stakeholders throughout their studies made professionals more confident in their research practices and increased their stakeholder involvement in following studies, which is why such programmes need to be offered more widely to professionals. Other possibilities for increasing patient–provider communication in research among a large number of stakeholders can also be the establishment of web-based surveys that aim to detect stakeholder priorities and ideas for the improvement of practices. This approach has already been implemented by Hall and colleagues in the James Lind Alliance Priority Setting Partnership (JLA PSP) for tinnitus [51] and by Mammarella and colleagues for Ménière’s disease, a condition related to tinnitus [52].

Connected to enhancing communication among stakeholders, the third action redirection was determined to be increasing interdisciplinarity within tinnitus practices. This can facilitate gaining a more holistic view of patients’ conditions and supports the collaborative exploration of, for instance, pathophysiological factors. Such interdisciplinary pathophysiological research has already been initiated for tinnitus [53]; however, it still needs to be more widely implemented. Another way of increasing interdisciplinarity is by organising interdisciplinary assemblies like the Tinnitus Research Initiative meeting [54]. Moreover, supporting interdisciplinary research projects such as the European School for Interdisciplinary Tinnitus Research (ESIT) [55] or the collaborative European TIN-ACT project [56] can further enable the exchange of information across disciplines. Within tinnitus treatments, more interdisciplinarity is needed from the onset of patients’ tinnitus condition onwards. Interdisciplinary clinics can facilitate the diagnosis of tinnitus as well as the treatment of possible comorbidities [57]. Since tinnitus causes, symptoms, or consequences can be similar to other medical or psychological conditions, professionals of various disciplines need to be involved in its diagnosis. This is to, for instance, let the cardiologist rule out arterial hyper- or hypotension [58], the neurologist rule out an acoustic neuroma [59], the dentist to rule out a temporomandibular joint disorder [57], the physical therapist to rule out cervicogenic somatic tinnitus [60], or the psychiatrist to rule out depressive disorders and suicidality [58]. Especially while no cure is available and considering patients’ high aim to find some kind of relief, this interdisciplinary approach can be a major aid in reducing their suffering and the severity of their condition [3]. Moreover, including disciplines such as psychology in these clinics can help at reducing physical barriers to seeking treatment that assists patients at emotionally coping with their condition [61]. 

Lastly, while searching for a cure, chronic patients should be offered more options to seek psychological support. With the current stigmas on such treatments, again, communications between patients and professionals need to be established in which possible reasons for stigmas and ways of addressing those are explored [62]. This still means that clinicians should aim to provide a general understanding of tinnitus and offering emotional support to patients; however, it should be openly communicated that further support for tinnitus management can be found in self-help groups or psychological treatments such as cognitive behavioural therapy (CBT) [59] or acceptance and commitment therapy (ACT) [63]. Moreover, occupational psychologists can also play an important role in helping patients reduce their suffering and enhancing their tinnitus management [64]. To reduce the barrier of seeking psychological support, Mehdi et al. [65] investigated and listed recommended smartphone applications that were specifically directed at helping tinnitus patients manage or, to a limited extent, treat their condition. With the more flexible use of such applications, they found patients felt less in need to seek in-person psychological or medical treatment, hence unburdening healthcare providers while simultaneously improving the way they dealt with their tinnitus [65]. Even if these technological applications are still in their infancy, their further development should be a primary focus of research to establish psychological care options for as many patients as possible. With this, more severely affected patients can then be filtered to see a psychologist and receive the support they need. Moreover, the chronic care model (CCM), which has already been successfully introduced to audiology but not yet to tinnitus treatments, could be implemented and tested for chronic tinnitus patients as it could help them better manage their condition over time with professional support [66].

### 4.4. Strengths and Limitations 

Due to the time-restricted nature of this research study, the sample for this pilot study was relatively small and data saturation could not be firmly established. Moreover, stakeholders such as ENTs, psychiatrists, and psychologists were not represented within the sample of this study. All participants had dealt with tinnitus for at least 5 years, the longest being 32 years. Patients had also operated as patient representatives prior to this study. Moreover, possibly due to the snowball sampling method used, participants held high educational degrees, with everyone holding a university degree, which was not intended by the researchers. As a result, the interests of lower-educated, newly established, not habituated, highly suffering, and less active patients were not represented within this sample. Lastly, while the PAR approach helped to include tinnitus stakeholders throughout the study and guide it in the direction they deemed important, stakeholder involvement and the exchange of feedback within the different research stages could still be increased too. Respecting these limitations, generalising the results of this study to the tinnitus population should be approached with caution, since only a small portion and group of stakeholders was involved within this project. Therefore, more studies on this issue should be conducted prior to the findings’ practical implementation in clinical and research settings.

Despite its limitations, this pilot study set another step towards increasing insights and communication among tinnitus stakeholders concerning their experiences and priorities for improving research and treatments. With the heterogeneity of experiences between and within stakeholder groups, a more holistic overview of issues connected to tinnitus practices could be established. To the best of the researchers’ knowledge, this is the first study to detect, compare, and stipulate tinnitus researcher, clinician, and patient perspectives while using the PAR approach to incorporate stakeholders throughout its progress. Some prior research projects, e.g., [47,48,49,51,67], did investigate stakeholder perspectives on tinnitus practices; however, they either did not include stakeholders throughout the study’s progress or did not address both treatment and research issues that need to be solved. Therefore, the work conducted within this pilot study, which includes these aspects, seems to be lacking in the current tinnitus literature. Moreover, the main action redirections established here illustrate the importance of increasing communication and the exchange of perspectives across tinnitus stakeholder groups. Therefore, this study also serves as evidence for the importance of the research conducted within this study and of its overall goal of including more voices of tinnitus stakeholders in the professional decision-making processes.

### 4.5. Recommendations for Future Research

This research can be seen as a pilot study for investigating tinnitus stakeholder perspectives on how to improve tinnitus research and treatment in the future. Future research could use this study as a general framework while establishing more longitudinal investigations including larger and more heterogeneous samples (e.g., in terms of professions, amount of time dealing with tinnitus, level of education, severity of suffering). Moreover, the PAR approach could be included in research even more, meaning that a larger number and a larger variety of tinnitus stakeholders should be involved in its planning, execution, and documentation. Following such research, the addressed action redirections need to be implemented in the actual context of professional tinnitus practices. It is desirable that multiple rounds of the PAR approach are implemented within such research as they can help to iteratively improve the action redirections based on constant interactions and reflections between stakeholders. To the best of the researchers’ knowledge, until now, there have been no studies to fulfil these recommendations for future research. However, a study related to these recommendations was conducted by Koch et al. [68], who implemented the PAR approach in a five-year study aimed at improving nursing clinicians’ wellbeing at the workplace. They found that participants reported significantly enhanced individual and community wellbeing at the end of the study [68], which illustrates the importance of implementing these suggestions into tinnitus research over time too. The findings of future similar studies could desirably help make tinnitus treatments and research more centred around stakeholder experiences. Based on these suggested future PAR studies, clinical guidelines such as the Flowchart for Patient Management by the Tinnitus Research Initiative, last updated in 2011 [58], and funded projects such as the JLA PSP, conducted in 2013 [51], could be adapted and updated. This could help to further negotiate the differences between stakeholders that were ascertained within this study. 

## 5. Conclusions 

This pilot study found that tinnitus stakeholders held partly similar, partly different expectations, needs, and perceived limitations on how to redirect tinnitus research and treatments. The main common goal was to achieve a pathophysiological understanding of tinnitus to develop a cure. Generally, it seemed that priorities and limitations of tinnitus research and treatments were often not communicated between patients and professionals, which might prevent many patients from improving their condition while being dissatisfied with the professional support offered. Moreover, professionals were facing issues that might not be solvable within solely one discipline, but which instead need an interdisciplinary approach (e.g., concerning pathophysiological research). 

Based on these findings, the main action priorities for redirecting tinnitus research and treatment are summarised as follows:

(1) Enhancing communication within and between stakeholders personally and professionally affected by tinnitus (e.g., by establishing patient participation sessions; offering codesign or cocreation workshops; in professional treatments).

Acting towards this priority can aid in reaching the other main priorities:

(2) Increasing the reflective use of patient involvement within professional practices (e.g., increasing professionals’ transparency about the risks associated with patient involvement such as reduced habituation to tinnitus perception; based on the provided information, letting patients decide whether to participate in research or not; offering workshops for professionals to feel more confident and skilled in including stakeholders throughout the research; using Option Grids in clinical encounters);
(3) Increasing interdisciplinarity in tinnitus research and treatment (e.g., interdisciplinary clinics or research projects to holistically investigate tinnitus’ pathophysiology and establish a cure; interdisciplinarity in the treatment of comorbidities);
(4) Reducing barriers to receiving psychological treatment while a cure is still not available (e.g., offering online treatment applications for tinnitus; talking about stigma towards psychological treatments with, e.g., medical professionals).

## Figures and Tables

**Table 1:** The two sets of six questions asked to tinnitus patients and professionals in the interviews.

Interview Question	Tinnitus Patients	Tinnitus Professionals
1	Could you give some information about yourself and your tinnitus background?	Could you give some information about yourself and your professional background?
2	What are your personal experiences with managing tinnitus?	What are your personal and professional experiences with tinnitus?
3	Would you say that you mainly self-manage your tinnitus condition or do you receive (e.g., psychological or medical) help to better deal with it?	According to you, which role does self-management play for tinnitus patients and their ways of dealing with tinnitus?
4	Do you generally feel confident about self-managing your condition?	Do you feel confident about dealing with patients who (are expected to) suffer from tinnitus?
5	What role does tinnitus research play for you and your way of managing your tinnitus condition?	What role does tinnitus research play for you and your way of practising your profession?
6	What are your personal hopes and suggestions for future tinnitus research and your wishes for yourself to improve your condition?	What are your personal hopes and suggestions for future tinnitus research, clinical management, and patients suffering from tinnitus?

## Data Availability

The data collected in this study are not made publicly available to ensure the anonymity and privacy of participants. Enquires about data access should be directed to the corresponding author.

## Abbreviations

ACT	Acceptance and Commitment Therapy
A.S.	co-author—Anneke Sools
CBT	Cognitive Behavioural Therapy
CCM	Chronic Care Model
COMiT’ID	Core Outcome Measures in Tinnitus
ENT	ear, nose, and throat
ESIT	European School for Interdisciplinary Tinnitus Research
GP	general practitioner
GS	co-author—Gerko Schaap
HRQoL	health-related quality of life
IQR	interquartile range
J.K.	first author—Julia Kajüter
J.P.S.	senior author—Jorge Piano Simões
JLA PSP	James Lind Alliance Priority Setting Partnership
Mdn	median
PA	patient
PAR	Participatory Action Research
PR	professional
TIN-ACT	Tinnitus Assessment, Causes, Treatments

## Appendix A. English Version of Probes for Tinnitus Patients as Shown on Presentation Slides

Question 1:

- **What** are the **symptoms** of your tinnitus?
- **When** did the symptoms **start**?
- Are the symptoms **consistent** or **irregular**?
- Have you ever received **professional treatment** for your tinnitus? (f.ex. by consulting an **ENT** doctor, **medical/psychological** care, …)
- **How burdened** do you feel by your condition?/How much does it **impact** your **daily life**?
- Next to your tinnitus, are there **other physical** or **psychological illnesses** that you suffer from?

Question 2:

- Easy/Difficult (Why? (e.g., (No) **support** systems, previous **experiences** with self-management in other conditions, part of **self-help groups**/receiving **professional treatment** that helps, good **knowledge** of clinicians/oneself on how to deal with it))

Question 3:

- If **Yes**: **Which** help do/did you get?
- **Why** do you (not) get additional help?
- Is there any **help** you would consider important for dealing with tinnitus that you **did not get yet**? **Which**? **Why** didn’t you get it yet? (f.ex. too expensive, too long waiting lists, too far away, fear that professionals will not take your condition seriously/have too little expertise to help you, …)
- Do you believe that (not) getting help **influenced** the extent to which you feel **confident** in **managing** your tinnitus condition? **Why**? If **Yes**: **How**?

Question 4:

- **Why** do you (not) feel confident about self-managing tinnitus?
- **What** would **help** you to feel (even) **more confident** about managing it? (f.ex. other support groups/more exchanging experiences with other affected individuals, more tailored tinnitus research/more updates on research, practical workshops, professional psychological treatment, …)

Question 5:

- Large/small/…; **Why**?
- Are there **topics** in tinnitus **research** that, according to you, are still **too largely ignored** even if they would **help** you to **better deal** with your condition? (f.ex. tinnitus management, cures, treatment options, reasons for tinnitus emergence, …); **Why** are they important to you/**How** would research in this area **help you** with dealing with tinnitus?
- Would you **inform** yourself about the **progress** of research projects if you knew that they **addressed personally relevant** topics? If **Yes**: **Which** topics would that be?
- Do you generally feel like tinnitus **research** could make you feel **more confident** in **managing** tinnitus? **Why**? **How**? **Which topics** would need to be addressed to make you feel more confident in self-managing tinnitus?

Question 6:

- finding a **cure**
- finding **better ways** of **supporting self-management** of tinnitus
- **more patient involvement** in research and treatment development
- increasing **professional support** and **expertise** to seek professional help with one’s tinnitus condition if needed

## Appendix B. English Version of Probes for Tinnitus Professionals as Shown on Presentation Slides

Question 1:

- What is your **profession**?
- **Since when** are you working in this field?
- Which **professional education** did you follow to be able to work in your occupation?
- Which **specialisations** or special **interests** do you have in your profession?
- In which fields related to your occupation do you already have **work experience**?

Question 2:

- How **often** are you **confronted** with tinnitus in your professional life?
- To what extent has tinnitus been **addressed** in your **professional education**?
- Are you **satisfied** with your **knowledge** of tinnitus or would you like to know more about it? If Yes: What/in **which areas** would you like to know more? **Why** do you **not know enough** about these areas yet (e.g., no advanced training on this issue, no research on it yet, lacking time to inform yourself)? What needs to **change** so that you **feel satisfied** with your knowledge of tinnitus?
- According to you, does tinnitus get the **attention** it **deserves/needs**?
- Based on your experience, **how** do tinnitus patients **experience** their condition? How **burdened** are they? What are their **biggest issues**?

Question 3:

- Why?
- What do you do when patients who (are expected to) suffer from tinnitus visit you? Is there a guideline you follow? Where does the focus of your treatment lie (e.g., offering professional help, psychological support, empowering patients to self-manage their condition)
- What do you think your patients need from you as a professional? Do you think you can fulfil these needs? How could you satisfy their needs even more? Do you think patients are satisfied with your way of treating tinnitus/Do you think your treatment is sufficient for helping them deal with it?
- According to you, what is currently missing in professional tinnitus treatment so that patients are/feel more supported when suffering from tinnitus?

Question 4:

- Large, small, … **Why?**
- **How** can, according to you, tinnitus **self-management** can be more **supported** (e.g., by you as a professional, psychological support, research, self-help groups, …)? How can **you help** patients at **becoming more confident** in self-managing their condition? How do you **already help patients** with **self-managing** tinnitus when they visit you?
- Do you think it is **more important** to **support** the **professional** treatment of tinnitus (e.g., medical or psychological treatment) or the **self-management** of affected individuals? Why?

Question 5:

- Large/small/…; **Why**?
- Do you think research can help you at **improving** your **treatment** of tinnitus patients? How? Research in which **area**? Which role does **patient and provider-oriented** research play in this respect?
- Do you think research can **help** patients **better deal** with/**self-managing** their condition? How? Which **topics** should be more addressed to reach this? Why? How could these topics be **approached** in **future research**?
- From your experience, to what **extent** do **professionals** dealing with tinnitus patients consider tinnitus **research** for their occupation? What could be **changed** in research or professional aspects so that **professionals** would take research **more into account** when treating tinnitus patients?

Question 6:

- finding a **cure**/successful **treatment**
- finding **better ways** of **supporting self-management** of tinnitus
- **more** patient and provider **involvement** in research and treatment development
- more **advanced training** on tinnitus topics
- **better preparation** in **studies** for professions dealing with tinnitus
- **changes** in professional tinnitus **treatment** (e.g., adapting diagnostic plans/guidelines and increasing their availability) and/or tinnitus **research** focuses
